# Low alpha-synuclein levels in the blood are associated with insulin resistance

**DOI:** 10.1038/srep12081

**Published:** 2015-07-10

**Authors:** Gerardo Rodriguez-Araujo, Hironori Nakagami, Yoichi Takami, Tomohiro Katsuya, Hiroshi Akasaka, Shigeyuki Saitoh, Kazuaki Shimamoto, Ryuichi Morishita, Hiromi Rakugi, Yasufumi Kaneda

**Affiliations:** 1 Division of Gene Therapy Science; 2 Department of Geriatric Medicine; 3 Department of Clinical Gene Therapy, Graduate School of Medicine, Osaka University, 2-2 Yamada-oka, Suita, Osaka, 565-0871, Japan; 4 Division of Vascular Medicine and Epigenetics, United Graduate School of Child Development, Osaka University, 2-1 Yamada-oka, Suita, Osaka, 565-0871, Japan; 5 Sapporo Medical University Hospital, Second Department of Internal Medicine.

## Abstract

Mutations in the protein alpha-synuclein (SNCA) have been linked to Parkinson’s disease. We recently reported that non-mutated SNCA enhanced glucose uptake through the Gab1-PI3 kinase-Akt pathway and elucidated its effects on glucose regulation. Here, we examined the association of SNCA with insulin resistance (IR), a condition that is characterized by decreased tissue glucose uptake. Our observations include those from a population study as well as a SNCA-deficient mouse model, which had not previously been characterized in an IR scenario. In 1,152 patients, we found that serum SNCA levels were inversely correlated with IR indicators—body mass index, homeostatic model assessment for IR (HOMA-IR) and immunoreactive insulin (IRI)—and, to a lesser extent, with blood pressure and age. Additionally, SNCA-deficient mice displayed alterations in glucose and insulin responses during diet-induced IR. Moreover, during euglycemic clamp assessments, SNCA knock-out mice fed a high-fat diet (HFD) showed severe IR in adipose tissues and skeletal muscle. These findings provide new insights into IR and diabetes and point to SNCA as a potential candidate for further research.

Metabolic syndrome is one of the most common findings in the clinic and affects millions of patients worldwide, resulting in a substantial burden on healthcare systems around the globe^1^,^2^,^3^. This syndrome is characterized by the presence of several risk factors and conditions that can precipitate the appearance of metabolic disorders, including hypertension, type 2 diabetes mellitus, obesity and dyslipidemias^4^. One of these conditions is insulin resistance (IR), which may lead to type 2 diabetes mellitus^3^.

The detailed mechanism of IR has yet to be elucidated. Among the hypothesized contributing factors are cytotoxic effects of palmitate in peripheral tissues^5^, Toll-like receptor-4-dependent NF-kB activation^6^, glycation damage^7^, and mitochondria dysfunction^8^. The first hypothesized contributing factor (palmitate), however, has drawn the most attention from researchers, giving rise to novel therapies such as G protein-coupled receptor (GPCR)-targeted therapies (i.e., GPR-40 agonists)^9^.

Alpha-synuclein (SNCA) is a protein richly expressed in neurons and hematopoietic tissues, and is present in serum^10^,^11^,^12^,^13^,^14^,^15^. Intriguingly, SNCA seems to function in some of the above-mentioned mechanisms underlying IR. SNCA interacts with lipids such as palmitate as a free acid-binding protein (FABP)-like transporter^16^, prevents glyoxalase I expression and glycation damage in neuron cells^17^, and has numerous extracerebral functions. SNCA was recently linked to glucose regulation as well as negative insulin secretion through K-channel modulation in beta cells of the pancreas^18^. The latter indicates that SNCA may have a role in decreasing hyperinsulinemia, which is observed in conditions such as IR. We also previously reported the function of SNCA in glucose metabolism *in vitro* and *in vivo* through a G-protein-coupled-receptor heterodimer lipo-phosphatidic acid receptor (LPAR2)- CD90^19^; however, how SNCA affects insulin is unclear. This question was the subject of our investigation.

Interestingly, we observed a previously unreported SNCA profile related to IR in both humans and rodents.

## Results

### Serum SNCA and IR in humans

We measured serum SNCA levels in a transversal study of a Japanese population from medical checkups in 2006 (Tanno-Subetsu in Hokkaido, *n* = 1152). To investigate differences between the patients’ serum SNCA levels and their metabolic profile, we categorized the data into 4 groups according to the serum SNCA levels (Grade 1, 13.4 ± 0.4 ng/dL; Grade 2, 35.2 ± 0.3 ng/dL; Grade 3, 52.8 ± 0.3 ng/dL and Grade 4, 95.1 ± 1.6 ng/dL). The Grade 1 and Grade 4 groups were the lowest- and highest-serum groups, respectively. We compared the differences between these two groups, as well as those between the intermediate groups (Grade 2 and 3). Each prespecified group had a similar number of subjects and included data on anthropometric and metabolic patient profiles (Table 1). After a preliminary analysis, we detected significant differences between SNCA grades, especially between the Grade 1 (lowest) and Grade 4 (highest) groups for serum immunoreactive insulin levels (IRI), homeostatic model assessment for IR (HOMA-IR), hematocrit (Hct) and systolic blood pressure values (SBP) via ANOVA (p < 0.0001). Next, these variables were analyzed to investigate correlations with the serum SNCA concentration itself (and not the grades) to avoid artifacts in the data analysis. Univariate linear regression models were independently fit for each study variable. The dependent variable in the model was the plasma SNCA concentration (ng/dL). The results from these models are given in Table 2. The variables with a significant association with the dependent variable at the 0.20 level were included in the multivariate model; thus, all the variables were included. To assess collinearity, tolerance factors were obtained for each variable in the multivariate model. The variables with tolerance factors <0.10 were examined further. HOMA-IR, IRI, hemoglobin (Hb), and hematocrit (Hct) had tolerance factors <0.10, and standard error estimates were much larger in the multivariate model than the univariate models. Pearson correlation coefficients were obtained for the associations between the four variables. HOMA-IR and IRI were strongly correlated (r = 0.92), as were Hb and Hct (r = 0.96). Furthermore, multivariate models were examined to determine which of these variables to keep in the final multivariate model. HOMA-IR and Hb were excluded from the initial multivariate model. The parameter estimates for the initial multivariate model, with tolerance factors, are provided in Table 3. To determine the final multivariate model, a stepwise selection was used. Both the entry and keep criteria in the model were set to an alpha of 0.20. The results from the final multivariate model are displayed in Table 4. The variables included in the model were age, BMI, IRI, diastolic blood pressure (DBP), and Hct. There was an inverse relationship between the dependent variable and age, BMI, IRI, and DBP, and a positive relationship between the dependent variables and Hct. These results are consistent with the results of the correlation analysis.

Because there is clinical interest in the association between HOMA-IR and outcome (SNCA concentration), the multivariate models were fit but excluded IRI and Hb. The parameter estimates for the initial multivariate model, with tolerance factors, are provided in Table 5. A stepwise selection was used to create the final multivariate model. Both the entry and keep criteria in the model were set to an alpha of 0.20. The results from the final multivariate model are displayed in Table 6. The variables included in the model were age, BMI, HOMA-IR, DBP, and Hct. There was an inverse relationship between the dependent variable and age, BMI, HOMA-IR, and DBP, and a positive relationship between the dependent variables and Hct. These results are consistent with those of the correlation analysis.

We found that Hb and Hct are positively correlated with SNCA levels, confirming the latter’s contribution to systemic SNCA serum levels as previously suggested^12^. Additionally, we found an inverse correlation with IR indicators (body mass index, HOMA-IR, and IRI) and a weaker correlation with DBP and age. These findings suggest that serum SNCA plays an important role in human metabolic disease, especially in insulin-glucose metabolism. Therefore, we further investigated SNCA’s metabolic implications in relation to IR and glucose metabolism.

### SNCAKO mice display impairment in glucose metabolism during diet-induced IR

**Figure 1:**
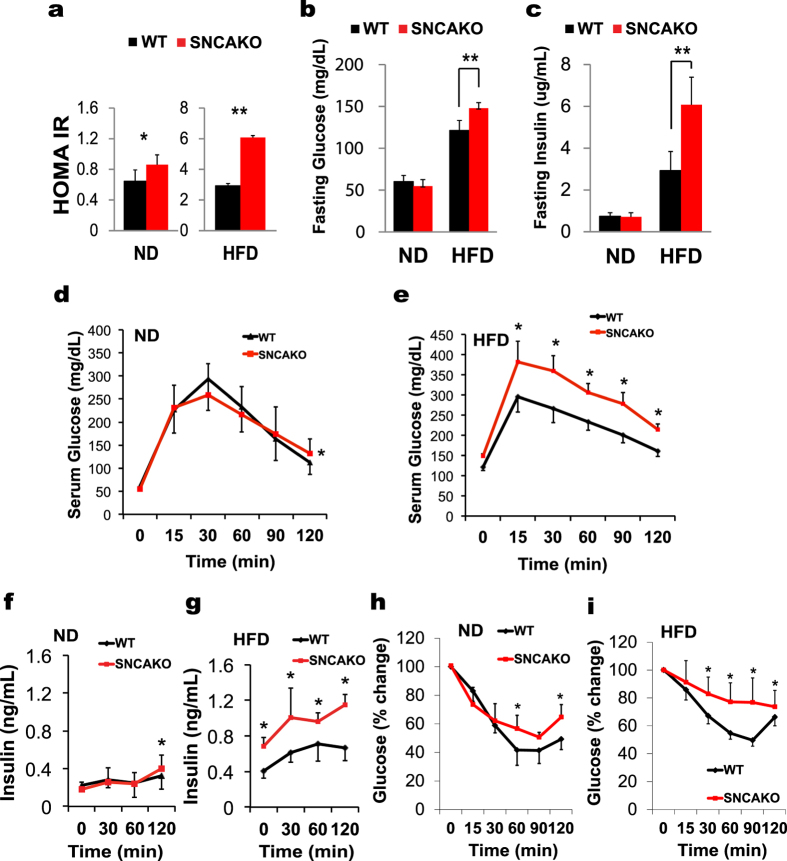
SNCAKO mice display disturbances in glucose metabolism. **(a)** Homeostatic model assessment index for insulin resistance (HOMA IR) in WT and SNCAKO mice after 5 weeks of a HFD. n = 10, each group. *P < 0.05 and **P < 0.01 via ANOVA. **(b)** Fasting blood glucose measurements in WT and SNCAKO mice fed a normal diet (ND) and a high-fat diet (HFD) for 5 weeks. n = 10, each group. **(c)** Fasting immunoreactive insulin measurements in WT and SNCAKO mice fed a ND or a HFD for 5 weeks. n = 10, each group. **(d)** Intraperitoneal glucose tolerance test (ipGTT, 2 g/kg) after 5 weeks of a ND in the WT and SNCAKO mice. n = 10, each group. **(e)** Intraperitoneal glucose tolerance test (ipGTT, 2 g/kg) after 5 weeks of a HFD in the WT and SNCAKO mice. n = 10, each group. **(f)** Serum insulin measurement (ELISA) during ipGTT in WT and SNCAKO mice fed a ND at 0, 30, 60 and 120 min. n = 10, each group.**(g)** Serum insulin measurement (ELISA) during ipGTT in WT and SNCAKO mice fed a HFD at 0, 30, 60 and 120 min. n = 10, each group. **(h)** Intraperitoneal insulin tolerance test (ipITT, 0.8 IU/kg) under a ND. n = 10, each group. **(i)** Intraperitoneal insulin tolerance test (ipITT, 0.8 IU/kg) after 5 weeks of a HFD. n = 10, each group. From (b) to (i): P < 0.05 was obtained for the area under the curve comparisons between the SNCAKO and WT groups (ANOVA test). In all the experiments: *P < 0.05; **P < 0.01.All histogram bars show the means ± s.d.

We used SNCA-deficient (SNCAKO) mice to perform the functional analysis of SNCA (Supplementary Fig. 1A and 1B). SNCAKO mice are viable and fertile, exhibit intact brain architecture, and have normal dopaminergic cell bodies, fibers and synapses^20^. We investigated the function of SNCA in Western diet-induced IR. We exposed SNCAKO mice to a high-fat diet (HFD). Under these conditions, the SNCAKO mice displayed a higher degree of IR compared with normal mice (WT) (C57/BL6), as indicated by an increased HOMA-IR index and higher levels of fasting blood glucose and serum insulin^21^,^22^,^23^,^24^ (Fig. 1A,C). Furthermore, SNCAKO mice displayed impairments in glucose and insulin responses as assessed via an intraperitoneal glucose tolerance test (ipGTT) and an intraperitoneal insulin tolerance test (ipITT), respectively. Additionally, SNCAKO mice presented increased insulinemia in ipGTT compared with their WT littermates, suggesting that SNCA plays an important role in glucose regulation and homeostasis during HFD-induced IR (Fig. 1D–I). Because SNCAKO demonstrated a poorer tissue insulin sensitivity profile during the ipITT and increased HOMA-IR, we decided to focus on IR in tissue. To address possible artifacts that may have affected our results, we performed all the experiments using animals with the same body weight and fat mass (Supplementary Fig. 1C and 1D).

**Figure 2:**
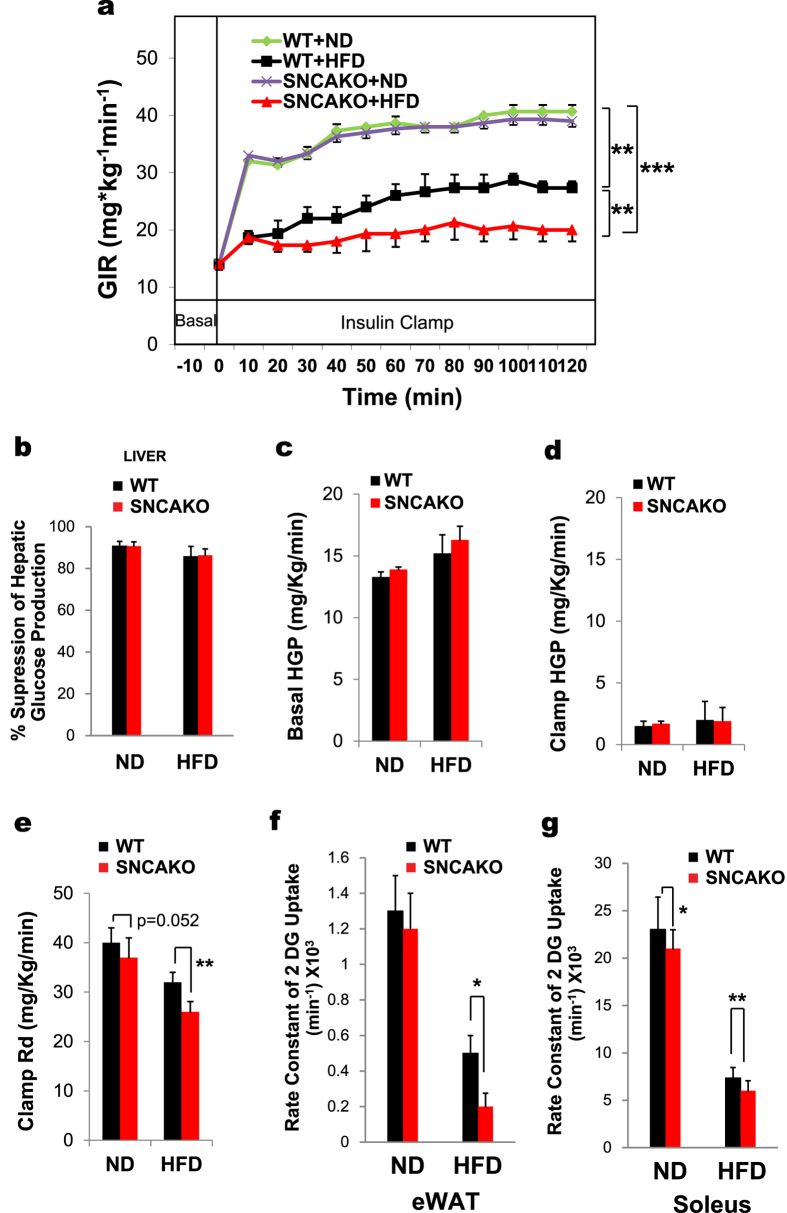
Hyperinsulinemic euglycemic clamp. SNCAKO mice display evident insulin resistance. **(a)** Hyperinsulinemic euglycemic clamp (HEC) in WT and SNCAKO mice fed a ND or a HFD (after 5 weeks). n = 5 each group. GIR, glucose infusion rate (mg*kg^−1^min^−1^). **(b)** Percentage suppression of hepatic glucose production (HGP) in WT and SNCAKO mice fed a ND or a HFD (after 5 weeks) during the HEC. n = 5 each group. **(c)** Basal HGP in WT and SNCAKO mice fed a ND or a HFD (after 5 weeks). n = 5 each group. **(d)** Clamp HGP in WT and SNCAKO mice fed a ND and a HFD (after 5 weeks). n = 5 in each group. **(e)** Clamp rate of disappearance indicating peripheral insulin resistance, especially in the HFD group. WT and SNCAKO mice fed a ND or a HFD (after 5 weeks). n = 5 in each group. **(f)** Glucose uptake in white adipose tissues in WT and SNCAKO mice fed a ND or a HFD during the HEC. Rate of [3H] deoxyglucose uptake in eWAT (epididymal white adipose tissue). n = 5 in each group. **(g)** Glucose uptake in the skeletal muscle of WT and SNCAKO mice fed a ND or a HFD during the HEC. Rate of [3H] deoxyglucose uptake in the soleus muscle. n = 5 in each group. In (a), each of the group comparisons were made at the end of the HEC using the area under the curve and WT ND group as the baseline response. In all the experiments: **P < 0.05; ***P < 0.001 via ANOVA test and Bonferroni’s correction when appropriate. Graph bars show the means ± s.d.

To determine whether SNCAKO mice are more sensitive to the development of IR under HFD, we applied hyperinsulinemic euglycemic clamps. Using this technique, we observed a significant increase in IR in the HFD-fed SNCAKO mice compared with the HFD-fed WT mice, with very modest changes (not statistically significant) between basal and insulin-hepatic glucose production (Fig. 2A,D). This IR was clearly noticeable in peripheral tissues, such as the adipose and skeletal tissues, of the SNCAKO mice (Fig. 2E,G). Taken together, our results indicate that SNCA plays an important role in diminishing insulin insensitivity in HFD-induced IR. Therefore, SNCA deletion results in a decreased tissue response to insulin; this is consistent with our initial findings in humans.

Finally, we measured the serum SNCA levels in diabetic mice. Interestingly, the serum SNCA level was decreased in db/db mice and also decreased with age (10, 24 and 96 weeks old) in mice (Supplementary Fig. 2A and 2B), similarly to our results in humans (Supplementary Fig. 2C). These results indicate that declining serum SNCA levels with age may contribute to the development of IR or diabetes.

## Discussion

This report elucidates the novel role of SNCA in IR. Ablation of SNCA exacerbates IR in tissues of rodents fed an HFD, and IR is also observed in patients with decreased SNCA serum levels. The higher the concentration of serum SNCA, the greater is the insulin sensitivity in both humans and rodents. SNCA demonstrated a clear benefit in terms of limiting IR in mice when exposed to HFD, as observed in our knock-out model and corroborated in the euglycemic clamp studies. The physiological function of glucose metabolism and regulation is to ensure effective energy substrate supplies for tissues. In the IR state, this mechanism is defective in transmitting signals to elicit sufficient energy substrate entry into the cell^25^,^26^. This status can be induced by exposure to saturated fats through a HFD^27^. Our report is the first to describe the use of SNCA knock-out mice for studying glucose metabolism in a pathological model (HFD IR model). We hypothesize that SNCA acts as a dynamic stress buffer to protect insulin signaling from saturated-fat-related IR and, additionally, as a promoter of glucose metabolism in tissues. *In vitro* evidence supports our hypothesis regarding lipids and indicates that SNCA could participate in the inhibition of fatty acid-dependent tissue toxicity because this protein can bind to lipids, has a high affinity for them and can interact with them through direct or indirect mechanisms^28^,^29^. Additionally, SNCA is important for glucose metabolism in tissues because it can elicit glucose uptake via the LPAR2-CD90 heterodimer (receptor), especially in adipose tissues and skeletal muscle^19^.

In humans, the SNCA serum levels and HOMA- IR or IRI were significantly and strongly correlated. Low levels of serum SNCA clearly displayed a higher HOMA- IR and insulin serum levels, which is congruent with the glucose metabolism profile of the SNCA knock-out mice. Interestingly, DBP, BMI and age were also correlated (modest correlation); this may indicate that SNCA is interacting with other risk factors of the metabolic disease together with HOMA-IR and IRI in humans. These SNCA interactions with conditions other than HOMA-IR and IRI require further research and will be the subject of future investigations by our research team.

The limitations of our population study include its transversal design. A prospective approach will give us a better understanding of the relationship between causality and serum SNCA over time as well as the cut-off points for normal serum SNCA levels. Additionally, because this study included only patients with Japanese ancestry, our findings cannot be generalized to other ethnicities. Another study in patients with metabolic disease or type 2 diabetes is needed to evaluate the possible associations between serum SNCA and disease severity or complications.

The strengths of our study include a large population, a broad spectrum of patient clinical characteristics, medical personnel with extensive experience in previous parallel clinical trials in the Tanno and Subetsu project, a comprehensive design (human and *in vivo* approaches) and a robust statistical analysis.

Currently, the narrow therapeutic arsenal against IR is a substantial challenge for physicians in attempts to reduce the risk of IR and/or type 2 diabetes onset in which tissue insulin response is greatly curtailed. As such, SNCA may provide new avenues to novel therapeutics in IR and diabetes.

## Materials and Methods

### Materials

We used recombinant SNCA (Atgen, South Korea) and insulin (Humulin-R100, Lilly, Japan). Anti-BSA (albumin) was purchased from Upstate Biotechnology (USA); all the other antibodies were purchased from Cell Signaling (USA).

### Human study

This was an observational cross-sectional study performed with 1,152 patients residing in Tanno and Subetsu in Hokkaido, Japan, in 2006^30^,^31^. The study was approved by Sapporo Medical University’s Institutional Review Board and in accordance with the Declaration of Helsinki. We included patients aged 20 years or more who were willing to participate in the study; they gave informed consent. We excluded severely sick patients with or without previous myocardial infarction or stroke as well as those with symptomatic cognitive disorders. Severely sick patients were defined as those who were in the terminal stages of chronic or acute diseases, such as type 2 diabetes mellitus (microvascular damage, i.e., retinopathy, diabetic foot, diabetic nephropathy), oncological diseases (solid or hematological tumors), sepsis, multiple organ dysfunction, cardiovascular disease, renal insufficiency or any other life-threatening disease that in the opinion of the principal investigator would have had a substantial impact on life expectancy, patient deambulation or ability to come to the clinic for medical checkups. These patients were excluded because they have very different pathophysiology that could interfere in the characterization of SNCA as a glucoregulator in a population study. The participating patients were enrolled consecutively in a prospective fashion; at the time of our investigation, this count was 1,152. A total of 1,152 patients were included and assessed for serum SNCA using the Human α-Synuclein Kit (Invitrogen, USA.). HOMA-IR was assessed in these patients using the following formula: Fasting glucose (mg/dL) × Immunoreactive insulin (μU/mL) / 405.

### Animal procedures

The experiments were approved by the Ethical Committee for Animal Experiments of the Osaka University Graduate School of Medicine. All the *in vivo* experiments were performed in compliance with Osaka University’s Animal Facility regulations. We used mice of the same body weight for the group comparisons. Serum insulin (Morinaga, Japan.), glucagon (R&D, USA.) and cortisol (Cayman Chemical Company, USA.) were quantified using ELISA.

## Methods

### Immunoblotting

The samples were processed with SDS-PAGE and transferred to PVDF membranes. Then, the membranes were blocked in 5% skim milk and incubated with their respective antibody. We assessed chemiluminescence using an Image Quant LAS4000 mini (GE, USA).

### Intraperitoneal glucose and insulin tolerance test (ipGTT and ipITT)

After 5 weeks of exposure to an HFD^32^ and after 8 hr of fasting, the mice were injected with glucose (2 g/kg) or insulin (0.8 IU/kg). Blood samples were extracted at 0, 15, 30, 60, 90 and 120 min.

### Glucose clamp

After cardiac catheterization and mice habituation, the mice were fasted for 5–6 h. Then, under euglycemic conditions, the WT and SNCAKO mice were assessed using radiolabeled tracers. Blood and tissue sampling was performed as previously reported (see Supplementary Materials)^33^,^34^.

### Statistical Analysis

The human data were analyzed using IBM SPSS Statistics 19. We used Stat View v5.0 for both the *in vivo* and i*n vitro* data. We presented the data as the means ± s.d. We used Student’s t test and ANOVA for statistical significance.

## Additional Information

**How to cite this article**: Rodriguez-Araujo, G. *et al.* Low alpha-synuclein levels in the blood are associated with insulin resistance. *Sci. Rep.* **5**, 12081; doi: 10.1038/srep12081 (2015).

## Supplementary Material

Supplementary Information

## Figures and Tables

**Table 1:** Anthropometric characteristics and metabolic profile in the studied population (n=1,152).

**Profile**	**SNCA Grade**				***P***
	**1**	**2**	**3**	**4**	
N	288	288	288	288	
Serum SNCA	13.4 ± 0.4	35.2 ± 0.3	52.8 ± 0.3	95.1 ± 1.6	<0.0001*
Age	64.9 ± 0.8	63.2 ± 0.8	62.8 ± 0.8	61.1 ± 0.8	0.0057*
Gender (Masculine)	40.8%	40.3%	40.6%	40.6%	0.9995
Height	155.5 ± 9.3	155.6 ± 9.3	156.6 ± 8.9	157.4 ± 9.1	0.1043
Weight	58.9 ± 10.5	58.2 ± 11.3	58.4 ± 11.3	58.2 ± 11.1	0.633
BMI	24.3 ± 3.7	23.9 ± 3.8	23.6 ± 3.3	23.4 ± 3.5	0.011*
Abdominal Circ.	87.0 ± 9.6	84.5 ± 10.0	85.0 ± 9.4	84.7 ± 9.8	0.126
Glucose	99.4 ± 20.1	96.9 ± 15.8	97.2 ± 19.4	96.2 ± 23.3	0.325
HbA1c	5.44 ± 0.66	5.33 ± 0.61	5.41 ± 0.65	5.36 ± 0.69	0.608
IRI	6.08 ± 3.9	5.36 ± 3.88	5.50 ± 4.62	4.77 ± 3.16	<0.0001*
HOMA IR	1.52 ± 1.0	1.35 ± 1.23	1.35 ± 1.26	1.16 ± 0.95	<0.0001*
RBC	442.4 ± 45.3	440.8 ± 47.6	450.6 ± 39.9	453.2 ± 40.3	0.0019*
Hb	13.49 ± 1.4	13.45 ± 1.4	13.74 ± 1.2	13.80 ± 1.3	0.0079*
Hct	41.4 ± 4.0	41.2 ± 4.0	42.1 ± 3.4	42.5 ± 3.5	0.0005*
WBC	5.41 ± 0.08	5.36 ± 0.08	5.19 ± 0.09	5.28 ± 0.09	0.273
Total Cholest	202.4 ± 34.0	197.4 ± 35.9	201.7 ± 31.7	203.6 ± 32.4	0.243
Triglycerides	106.7 ± 50.6	111.3 ± 72.1	105.0 ± 74.8	101.7 ± 69.6	0.628
HDL	52.4 ± 15.4	53.0 ± 14.2	54.2 ± 13.6	55.6 ± 13.1	0.011
SBP	143.5 ± 22.9	138.9 ± 23.2	137.7 ± 23.3	134.9 ± 21.2	<0.0001*
DBP	78.6 ± 10.9	77.8 ± 12.6	76.3 ± 11.2	75.3 ± 10.7	0.0023*
TP	7.32 ± 0.37	7.33 ± 0.39	7.34 ± 0.39	7.35 ± 0.39	0.0186*
Uric Acid	5.33 ± 1.34	5.09 ± 1.25	5.14 ± 1.30	5.13 ± 1.36	0.1963
BUN	15.8 ± 4.7	15.4 ± 4.0	15.3 ± 4.3	15.3 ± 3.6	0.676
Creatinine	0.68 ± 0.20	0.66 ± 0.14	0.71 ± 0.63	0.66 ± 0.15	0.3078
AST	26.1 ± 10.2	27.9 ± 13.3	24.0 ± 8.2	24.0 ± 8.2	0.0413*
ALT	23.8 ± 13.2	25.1 ± 17.2	22.4 ± 13.2	22.4 ± 13.3	0.1258

Serum SNCA, alpha synuclein serum level (ng/dL); Age (years); Height (cm); Weight (kg); BMI, Body Mass Index (kg/cm2); Abdominal Circ, abdominal circumference (cm); Glucose, fasting blood glucose (mg/dL); HbA1c, glycated hemoglobin (%); IRI, Immunoreactive insulin (μU/mL); HOMA IR, Homeostatic model assessment insulin resistance (HOMAIR Units); RBC, red blood cells count (X10,000 cells/μL); Hb, hemoglobin (g/dL); Hct, hematocrit, Hct (%); WBC, white blood cells count (X1,000 cells/μL); Total Cholest, total cholesterol (mg/dL); Triglycerides (mg/dL), HDL, high density lipoproteins concentration (mg/dL); SBP, systolic blood pressure (mmHg); DBP, diastolic blood pressure (mmHg); TP, total serum protein (g/dL); Uric acid (mg/dL); BUN, blood urea nitrogen (mg/dL); Creatinine (mg/dL); AST, aspartate aminotranferase (U/L); ALT, alanine aminotransferase (U/L). Significant differences were assesed by ANOVA test. ± SD, plus/minus standard deviation, *P < 0.0019.

**Table 2:** Univariate Linear Regression Model Parameter Estimates.

**Variable**	**Estimate (β)**	**StdErr**	**tValue**	**P-value**
Age	−0.27775	0.07460	−3.72	0.0002
BMI	−0.95687	0.27199	−3.52	0.0005
HOMA IR	−4.85829	0.88828	−5.47	<0001
IRI	−1.46830	0.25754	−5.70	<0001
Glucose	−0.09380	0.04657	−2.01	0.0442
SBP	−0.17848	0.04333	−4.12	<0001
DBP	−0.30613	0.08728	−3.51	0.0005
RBC	0.07289	0.02294	3.18	0.0015
Hb	1.91436	0.72883	2.63	0.0087
Hct	0.95129	0.26084	3.65	0.0003

Dependent Variable: SNCA concentration (ng/dL).

Univariate linnear regression model for each study variable. HOMA- IR displayed high β values (−4.85, p < 0.0001) together with IRI (−1.46, p < 0.0001). Hb and Hct were also correlated with SNCA serum levels in a lower degree. SNCA, alpha synuclein; Age (years); BMI, Body Mass Index (kg/cm2); HOMA IR, Homeostatic model assessment for insulin resistance (HOMAIR Units); IRI, Immunoreactive insulin (μU/mL); Glucose, fasting blood glucose (mg/dL); SBP, systolic blood pressure (mmHg); DBP, diastolic blood pressure (mmHg); RBC, red blood cells count (X10,000 cells/μL); Hb, hemoglobin (g/dL); Hct, hematocrit, Hct (%). n = 1152.

**Table 3:** Initial Multivariate Linear Regression Model Parameter Estimates.

**Variable**	**Estimate (β)**	**StdErr**	**tValue**	**P-value**	**Tolerance**	**Variance Inflation**
Intercept	39.91342	13.53136	2.95	0.0032	—	—
Age	−0.16249	0.08982	−1.81	0.0707	0.66026	1.51455
BMI	−0.38189	0.31258	−1.22	0.2221	0.72602	1.37738
IRI	−1.36687	0.28699	−4.76	<0001	0.77726	1.28656
Glucose	−0.04843	0.04741	−1.02	0.3072	0.91121	1.09744
SBP	−0.01448	0.06661	−0.22	0.8280	0.40433	2.47325
DBP	−0.29349	0.12065	−2.43	0.0151	0.49757	2.00978
RBC	−0.00454	0.04609	−0.10	0.9216	0.24473	4.08608
Hct	1.59311	0.51290	3.11	0.0019	0.25240	3.96189

Dependent Variable: SNCA concentration (ng/dL).

Parameter estimates for the initial multivariate model using IRI. HOMA-IR was excluded from this model to avoid collinearities. SNCA, alpha synuclein; Age (years); BMI, Body Mass Index (kg/cm2); IRI, Immunoreactive insulin (μU/mL); Glucose, fasting blood glucose (mg/dL); SBP, systolic blood pressure (mmHg); DBP, diastolic blood pressure; RBC, red blood cells count (X10,000 cells/μL); Hct, hematocrit, Hct (%); (mmHg). n = 1152.

**Table 4:** Final Multivariate Linear Regression Model Parameter Estimates.

**Variable**	**Estimate (β)**	**StdErr**	**tValue**	**P-value**	**Tolerance**	**Variance Inflation**
Intercept	37.50949	13.04383	2.88	0.0041	—	—
Age	−0.18183	0.07438	−2.44	0.0147	0.96132	1.04024
BMI	−0.42207	0.30801	−1.37	0.1709	0.74649	1.33960
IRI	−1.39992	0.28442	−4.92	<0001	0.79013	1.26561
DBP	−0.30748	0.09060	−3.39	0.0007	0.88103	1.13504
Hct	1.52374	0.27512	5.54	<0001	0.87582	1.14179

Dependent Variable: SNCA concentration (ng/dL).

Final multivariate linear regression model using IRI. In this model, stepwise selection was used. HOMA-IR was excluded from this model to avoid collinearities. Both the entry and keep criteria into the model was set at the alpha level of 0.20. SNCA, alpha synuclein; Age (years); BMI, Body Mass Index (kg/cm2); IRI, Immunoreactive insulin (μU/mL); Hct, hematocrit, Hct (%); DBP, diastolic blood pressure (mmHg). n = 1152.

**Table 5:** Initial Multivariate Linear Regression Model Parameter Estimates.

**Variable**	**Estimate (β)**	**StdErr**	**tValue**	**P-value**	**Tolerance**	**Variance Inflation**
Intercept	34.36649	13.91504	2.47	0.0137	—	—
Age	−0.16930	0.08993	−1.88	0.0600	0.66018	1.51474
BMI	−0.45627	0.30940	−1.47	0.1406	0.74261	1.34660
HOMA IR	−4.95366	1.10339	−4.49	<0001	0.62547	1.59879
Glucose	0.03628	0.05330	0.68	0.4962	0.72269	1.38373
SBP	−0.01575	0.06671	−0.24	0.8134	0.40408	2.47477
DBP	−0.29285	0.12082	−2.42	0.0155	0.49728	2.01095
RBC	−0.00632	0.04613	−0.14	0.8911	0.24482	4.08458
Hct	1.58563	0.51345	3.09	0.0021	0.25241	3.96181

Dependent Variable: SNCA concentration (ng/dL).

Parameter estimates for the initial multivariate model using HOMA-IR. IRI was excluded from this model to avoid collinearities. SNCA, alpha synuclein; Age (years); BMI, Body Mass Index (kg/cm2); HOMA IR, Homeostatic model assessment for insulin resistance (HOMAIR Units); Glucose, fasting blood glucose (mg/dL); SBP, systolic blood pressure (mmHg); DBP, diastolic blood pressure; RBC, red blood cells count (X10,000 cells/μL); Hct, hematocrit, Hct (%); (mmHg). n = 1152.

**Table 6:** Final Multivariate Linear Regression Model Parameter Estimates.

**Variable**	**Estimate (β)**	**StdErr**	**tValue**	**P-value**	**Tolerance**	**Variance Inflation**
Intercept	36.22095	13.13491	2.76	0.0059	—	—
Age	−0.16978	0.07445	−2.28	0.0228	0.96098	1.04061
BMI	−0.47702	0.30565	−1.56	0.1189	0.75931	1.31698
HOMA IR	−4.60500	0.97431	−4.73	<0001	0.80043	1.24932
DBP	−0.31489	0.09063	−3.47	0.0005	0.88188	1.13394
Hct	1.54805	0.27585	5.61	<0001	0.87261	1.14598

Dependent Variable: SNCA concentration (ng/dL).

Final multivariate linear regression model using HOMA- IR In this model, stepwise selection was used. IRI was excluded from this model to avoid collinearities. Both the entry and keep criteria into the model was set at the alpha level of 0.20. SNCA, alpha synuclein; Age (years); BMI, Body Mass Index (kg/cm2); HOMA IR, Homeostatic model assessment for insulin resistance (HOMAIR Units); DBP, diastolic blood pressure (mmHg); Hct, hematocrit, Hct (%). n=1152.
