# Supplementary material for: Low alpha-synuclein levels in the blood are associated with insulin resistance
Source: Sci Rep. 2015 Jul 10;5:12081. doi: 10.1038/srep12081 (PMC4498217; doi:10.1038/srep12081)
Supplement: Supplementary Information [file srep12081-s1.pdf]

Supplementary Materials

Experimental Procedures.

Low alpha-synuclein levels in the blood are associated with Insulin Resistance.

Gerardo Rodriguez-Araujo[1], Hironori Nakagami[2],Yoichi Takami[4], Tomohiro Katsuya[3], Hiroshi Akasaka[5], Shigeyuki Saitoh[5], Kazuaki Shimamoto[5], Ryuichi Morishita[3], Hiromi Rakugi[4] & Yasufumi Kaneda[1].

[1]Division of Gene Therapy Science, [3]Department of Clinical Gene Therapy, [4]Department of Geriatric Medicine, Graduate School of Medicine, Osaka University, 2-2 Yamada-oka, Suita, Osaka, 565-0871, Japan.
[2] Division of Vascular Medicine and Epigenetics, United Graduate School of Child development, Osaka University, 2-1 Yamada-oka, Suita, Osaka, 565-0871, Japan.
[5]Sapporo Medical University Hospital, Second Department of Internal Medicine

Supplemental Experimental Procedures

Human protocol.

Study Design. This study is a transversal and observational trial. It was performed in 1,152 patients residing in Tanno and Subetsu towns in Hokkaido, Japan in 2006. The primary aim of this study was to investigate for existent associations between serum SNCA levels and metabolic profile in the general population. Further specific objective was to investigate probable associations between serum SNCA levels and metabolic disease (insulin resistance or metabolic syndrome). Part of the study design of this protocol was published in a parallel study protocol elsewhere[1]. Initial anthropometric parameters and blood pressure were measure by physicians in their respective medical center. Blood pressure measurements were obtained using an automated sphyngomanometer (HEM-907, Omron Corporation, Japan). Later, blood samples obtention and screening were performed in all patients. Serum SNCA was quantified in all samples by enzyme-linked immunosorbent assay (ELISA) from Invitrogen, USA. Immunoreactive insulin (IRI) was obtained by radioimmunoassay method. Glycated hemoglobin (HbA1c) was measured by latex coagulation method. Triglycerides were measured by glycerol-3-phosphate-oxidize-DAOS method.

Insulin resistance was assessed in patients by the homeostatic model assessment index for insulin resistance (HOMA IR), obtained as follows:

$$\textbf{HOMA IR} = \frac{\text{Fasting Glucose (mg/dL) X Immunoreactive Insulin (}\mu\text{U/mL)}}{405}$$

Metabolic syndrome was defined in accordance with the Asian modified criteria of ATPIII guidelines [2].

Profile	Value
Abdominal Circumference	≥90 cm in men ≥80 cm in women
Fasting Triglycerides	≥150 mg/dL
HDL	<40 mg/dL in men <50mg/dL in women
Blood pressure	≥130/≥85 mmHg
Fasting glucose	≥110 mg/dL

.

This protocol was performed in collaboration with Sapporo University with the approval of the Institutional Review Board of Sapporo Medical University. Informed consent was obtained from all patients before enrollment in this study.

In vitro and *in vivo* Supplemental Experimental Procedures.

Animal procedures. The experiments were approved by the Ethical Committee for Animal Experiments of the Osaka University Graduate School of Medicine. The mice had free access to water and food during the experimental periods. Ten week old male C57/BL6 (WT) and db/db (from Clea, Japan) as well as SNCAKO (from The Jackson Laboratory) were handled in compliance with Osaka University's Animal Facility regulations. They were conserved under light-dark, no pathogen but non sterile conditions. For group comparisons, in all experiments we used mice under same body weight.

Immunobloting. Samples were run in multigradient gel (Multigel II Mini, Cosmo Bio). Transference was performed using PVDF transfer membranes (Amersham Hybond, GE Health Care). Membranes were blocked using 5% skim milk (Nacalai Tesque) in PBS-T (tween 0.05%) for 1 hr and incubated overnight with primary antibody. Then, membranes were washed with PBS-T three times and incubated with HRP linked IgG (Rabbit/Mouse, GE Health Care). Subsequent, washing with PBS-T three times was performed and then ECL plus WB detection system (GE Health Care) was added to membranes and incubated for 10 min. Later, membranes were exposed to a high performance chemiluminescence film (Amersham, GE healthcare) and then developed in a Medical Film Processor (Fuji Film) or an Image Quant LAS4000 mini (GE) using manufacturer's manual.

For immunoblot analysis of serum samples, 10 μL of plasma was mixed with sample buffer and run by SDS Page for posterior blotting.

Antibodies. Rabbit antibodies for Alpha Synuclein IF was purchased from Cell Signaling. Monoclonal mouse beta actin was purchased from Sigma.

Intraperitoneal glucose and insulin tolerance test [3] (ipGTT and ipITT), WT and SNCAKO mice were fed with high fat diet (HFD; fat 20.4%, protein 33.2%, carbohydrates 46.4%). Experiments were performed after 5 weeks of HFD in the respective group. Before the experiments mice were fasted overnight (8 hr) and water was supplied *ad libitum*. Then, glucose (2 g/kg) and insulin 0.8 IU/kg (Humulin R, Lilly) were injected in mice respectively and blood glucose levels were measured using a Glucometer AS-R (SKK, Japan) at the timelines of 0, 15, 30, 60, 90 and 120 min. Extra blood sample (20 μl) was extracted at 0, 30, 60,

120 min to evaluate plasma insulin by ELISA system (Mouse insulin, Morinaga, Japan).

Cardiac catheterism. Mice weighting 24 gr were anesthetized intraperitoneally with medetomidine 0.3 mg/kg, midazolam 0.8 mg/kg and butorphanol 1 mg/kg. The catheterism procedure is described previously [4]. Briefly, installation of silicon cardiac catheter was performed in mice using the right jugular vein. Catheter was fixed and heparinized. One to three days later, the experiments were performed. During that time the mice were habituated to procedure's handling. Intravascular localization of the catheter was confirmed postmortem in all the mice after the experiment was finished.

Hyperinsulinemic euglycemic clamp [5,6]**.** Briefly, as previously described, mice were submitted to cardiac catheter installation and habituated to handling for 3 days. The mice were fasted for 5- 6 h before the experiment. Then, baseline blood samples were extracted from the cardiac catheter and priming solution was infused. A priming dose was 1 μCi of [3-3H] glucose infused at a rate of 20 μl/min for 1 min. Followed by a continuous infusion of [3-3H] glucose at 0.05 μCi/min (1 μl/min) for 90 min (equilibration period). Then, we infused insulin starting from a rate of 4 mU•kg-1•min-1 and subsequently adjusted to achieve a final blood glucose target of 145 mg/dL in the studied mice. Blood samples were taken every 10 min starting from min 0 until min 120. After that, we performed the tissue specific glucose uptake in eWAT and soleus muscle as described previously. We used organic solvent (Solvable, Perkin Elmer) and scintillation solutions (Ultima

Gold, Perkin Elmer) were added to the LSC vial (Tissues and blood samples) to be assessed by a Wallac 1409 Liquid Scintillation Counter.

Software and Data Analysis. Human data was analyzed using IBM SPSS Statistics 19. *In vivo* and i*n vitro* data was statistically analyzed using Stat View v5.0. We presented data as means ± s.d. We used student's t test and ANOVA analysis for statistical significance. Bonferroni's test was used for multiple comparisons when appropriate. Immunoblottings and pictures were analyzed or visualized with Adobe Photoshop 7.0.1, Image J v1.37c and Adobe Illustrator CS5.

SUPPLEMENTAL REFERENCES

1 Chiba, M. *et al.* Associations of metabolic factors, especially serum retinol-binding protein 4 (RBP4), with blood pressure in Japanese--the Tanno and Sobetsu study. *Endocr J* **57**, 811-817 (2010).

2 Heng, D. *et al.* Modification of the NCEP ATP III definitions of the metabolic syndrome for use in Asians identifies individuals at risk of ischemic heart disease. *Atherosclerosis* **186**, 367-373 (2006).

3 Begriche, K. *et al.* Beta-aminoisobutyric acid prevents diet-induced obesity in mice with partial leptin deficiency. *Obesity (Silver Spring)* **16**, 2053-2067 (2008).

4 Shiuchi, T. *et al.* Involvement of bradykinin and nitric oxide in leptin-mediated glucose uptake in skeletal muscle. *Endocrinology* **142**, 608-612 (2001).

5 Shiuchi, T. *et al.* Hypothalamic orexin stimulates feeding-associated glucose utilization in skeletal muscle via sympathetic nervous system. *Cell Metab* **10**, 466-480 (2009).

6 Ayala, J. E. *et al.* Hyperinsulinemic-euglycemic clamps in conscious, unrestrained mice. *J Vis Exp* **57**, 3188 (2011).

Supplementary Fig 1

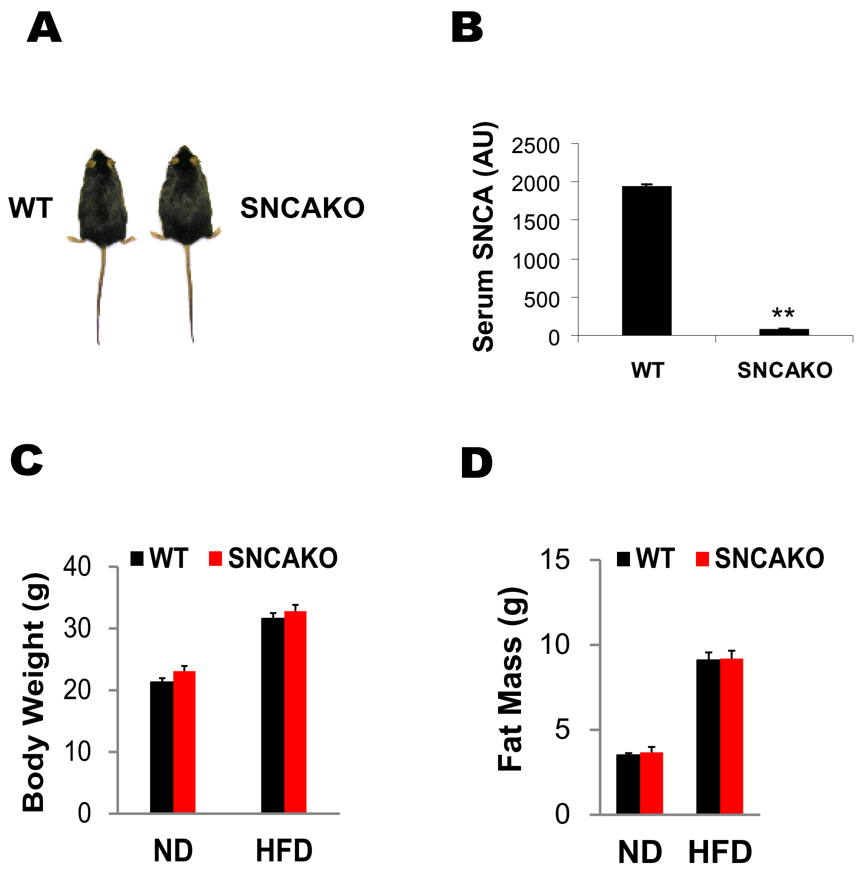

Supplementary Fig 1.

(A) Wild type (WT) and alpha synuclein knock out (SNCAKO) mice appearance. SNCAKO mice are fertile and viable.

(B) Serum SNCA assesment in WT and SNCAKO mice. Serum albumin was used as loading control.

(C) Body weight of WT and SNCAKO mice under normal chow (ND) and high fat diet (HFD) at 12 weeks and 17 weeks old. Both littermates were exposed to HFD for 5 weeks. n=10 each group.

(D) Fat mass in grams of WT and SNCAKO mice under normal chow (ND) and high fat diet (HFD) at 12 weeks and 17 weeks old. Both littermates were exposed to HFD for 5 weeks. n=10 each group.

Supplementary Fig 2

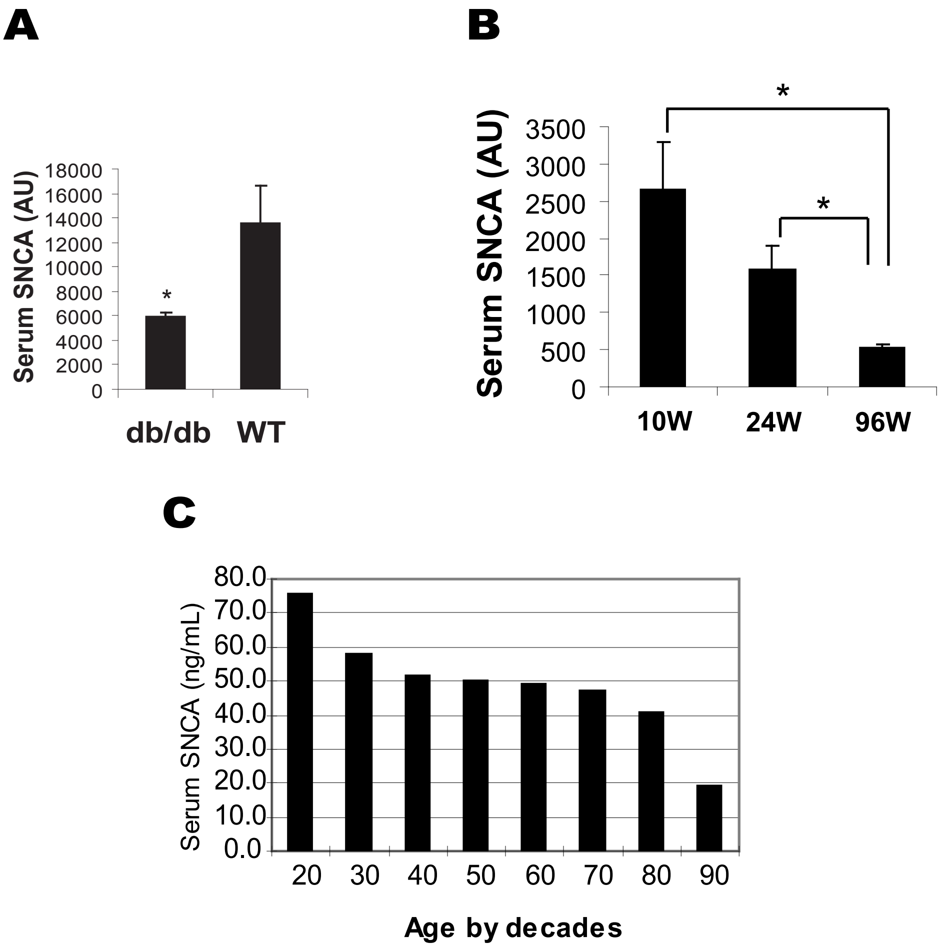

Supplementary Fig 2.

(A) Diabetic mice displayed low serum SNCA levels. Immunoblot analysis of 10 μL of blood from db/db and WT mice. Serum albumin was used as loading control. n=4 each group.

(B) 10 μL of WTmouse plasma was blotted to detect serum SNCA. 10W, 10 weeks old; 24W, 24 weeks old; 96W, 96 weeks old mice. Serum albumin is used as loading control. n=2 each group.

(C) Serum SNCA was decreased by aging in the population study (human). Serum SNCA level showed a decreasing pattern with age in Japanese population. n=1,152 patients serum samples. 20 = 20-29 years old, 30 = 30-39 years old, 40 = 40-49 years old, 50 = 50-59 years old, 60 = 60-69 years old, 70 = 70-79 years old, 80 = 80-89 years old and 90 = 90-99 years old.
